# Omalizumab as a corticosteroid‐sparing agent in the treatment of bullous pemphigoid

**DOI:** 10.1111/dth.15946

**Published:** 2022-10-31

**Authors:** Camilla Vassallo, Anita Somenzi, Mara De Amici, Stefania Barruscotti, Valeria Brazzelli

**Affiliations:** ^1^ Dermatology Clinic Fondazione IRCCS Policlinico San Matteo Pavia Italy; ^2^ Immuno‐Allergology Laboratory, Clinical Chemistry Unit Fondazione IRCCS Policlinico San Matteo Pavia Italy; ^3^ Department of Clinical‐Surgical, Diagnostic and Pediatric Science University of Pavia Pavia Italy

**Keywords:** bullous pemphigoid, comorbidities, corticosteroid‐sparing, omalizumab

## Abstract

Bullous pemphigoid (BP) is the most common autoimmune blistering skin disease, characterized by the development of autoantibodies against hemidesmosomal components BP180 and BP230. The mainstay of therapy is topical and systemic corticosteroids (CS) and immunosuppressors. As this pathology mainly involves the elderly, subjects often have numerous comorbidities that influence the clinical management. Omalizumab is a recombinant humanized monoclonal anti‐IgE antibody which has recently emerged as a promising treatment for BP in patients for whom CS are contraindicated or conventional treatments have failed to control the disease. For this study, we selected five patients who presented with corticosteroid‐dependent BP with a contraindication to the use of other immunosuppressive treatments. The objectives of our study were to evaluate the effectiveness of omalizumab in controlling BP and allowing to decrease the dosage of systemic CS, assessing the effects of omalizumab on the clinical manifestations and the titers of circulating anti‐BP180 and BP230 antibodies, IgE and eosinophils. A reduction in the dose of systemic CS was possible in 100% of the patients and complete resolution of the clinical picture was seen in 100% for skin lesions and in 40% for pruritus. A reduction of circulating IgE was found in 40%, anti‐BP180 and BP230 IgGs were decreased in 60% and eosinophils in 80%.

## 1 INTRODUCTION

Bullous pemphigoid (BP) is the most common autoimmune blistering skin disease, characterized by the development of autoantibodies against hemidesmosomal anchoring components BP180 and BP230. The mainstay of therapy is topical and systemic corticosteroids (CS) and immunosuppressors. Mortality is reported to be up to 40% in the first year.^1^, ^2^ As this pathology mainly involves the elderly, patients often have numerous comorbidities that influence the clinical management and the choice of drugs.

Omalizumab is a recombinant humanized monoclonal anti‐IgE antibody, recently emerged as a promising adjuvant treatment for BP, following the evidence that IgE autoantibodies also have a relevant role in the pathogenesis of the disease.^3^, ^4^

The use of this monoclonal antibody has initially been proposed in the therapy of BP in 2009 and since then, numerous case series and reports have been published.^5^, ^6^

Our aim was to evaluate the effectiveness of omalizumab treatment in reducing signs and symptoms of BP, thus allowing to decrease the dosage of systemic CS.

## 2 METHODS

We retrospectively evaluated all the patients receiving treatment for BP in the Autoimmune Bullous Disease Department of the Dermatology Clinic, University of Pavia, IRCCS Policlinico San Matteo Foundation; among 222 subjects evaluated for BP in the last 10 years, we selected five patients who presented with corticosteroid‐dependent BP, with a contraindication to the use of other immunosuppressive treatments and who could instead benefit from the use of a safer and selectively‐acting agent like omalizumab.

We performed a retrospective chart review and obtained information from patients' files and laboratory reports.

The diagnosis of BP was based on the presence of bullous and/or urticarial cutaneous lesions confirmed by histological examination and direct immunofluorescence on skin biopsies. The titers of anti‐BP180 and BP230 at the time of diagnosis were also obtained from the medical records, using as a threshold value for positivity >20 U/mL (EuroImmun, Lubeck, Germany). Indirect immunofluorescence with also salt‐split skin assay was performed in two out of five patients (EuroImmun, Lubeck, Germany). One patient had negative antibody values against BP180 and BP230: the diagnosis of BP was possible through direct and indirect immunofluorescence, in particular salt split skin assay revealed IgG deposits at the roof of the blister. Furthermore, the antibodies against collagen VII were within the reference range. The absence of circulating autoantibodies could be explained by the fact that the ELISA kit used identifies only the immunodominant NC16A domain. Our patient could be among those described to have autoantibodies only against regions of BP180 outside of NC16A.

The variables studied for each patient were age and sex, pharmacological treatment received for BP and comorbidities. To assess the effects of the adjuvant therapy with omalizumab we studied eosinophilia, total IgE levels, BP180 and BP230 autoantibodies and dosage of systemic corticosteroid therapy, both before and during omalizumab treatment. Due to the severity of the clinical picture and side effects of systemic steroid therapy, three patients have used immunosuppressive therapies (i.e., methotrexate) as adjuvant treatment to reduce the dosage of prednisone. Immunosuppressive therapy was discontinued due to side effects. The other two patients had multiple comorbidities and it was not possible to introduce immunosuppressive therapies.

To assess the clinical response to this type of treatment the ABSIS score^7^ was used, while to assess the pruritus we used the VAS scale,^8^ both of these variables were evaluated after 6 months of treatment.

Omalizumab was administered as a single subcutaneous (SC) dose of 300 mg, once every 4 weeks, following the chronic idiopathic urticaria protocol according to which a cycle corresponds to 6 months followed by a suspension of 8 weeks before a new cycle can be performed.^9^ The patients in our study had received treatment with omalizumab over a time‐frame of 3 years and 6 months, from January 2018 to June 2021.

**FIGURE 1:**
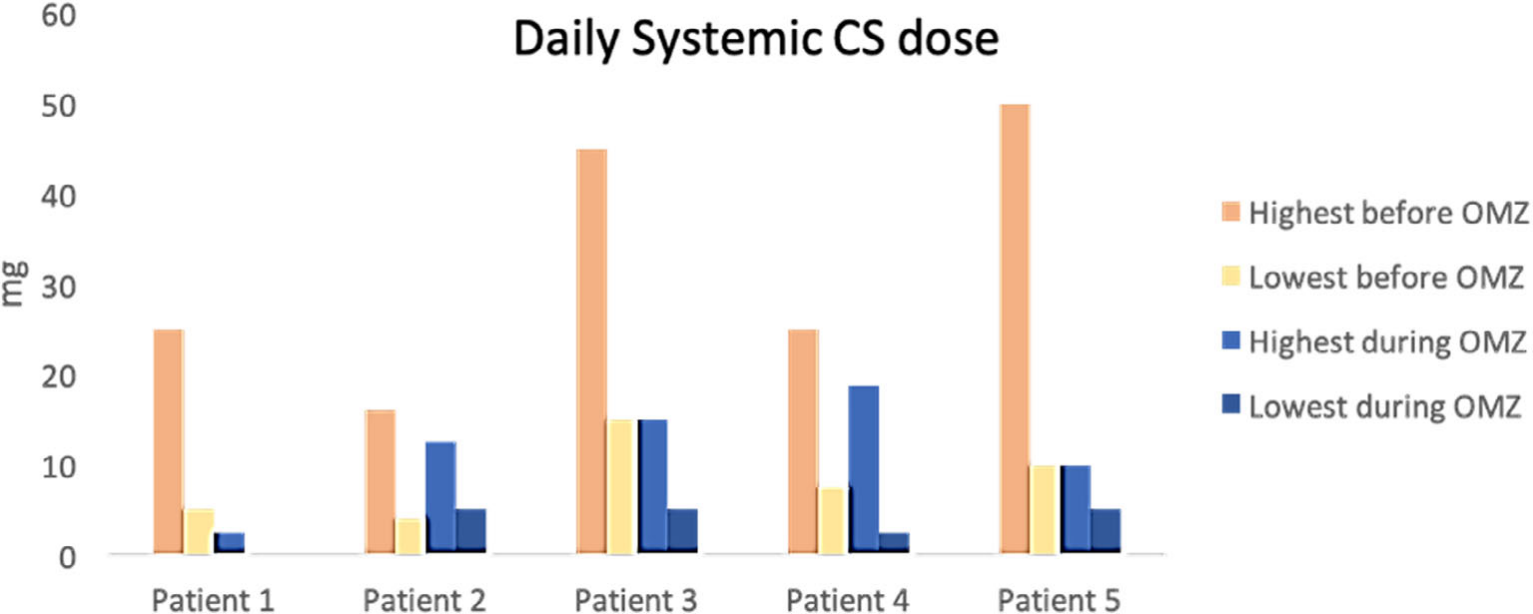
Course of systemic steroid therapy (prednisone mg/day) before and after therapy with omalizumab (OMZ)

Our patients' data are displayed in Tables 1, 2, and Figure 1.

**TABLE 1:** Patients' past medical history and information regarding bullous pemphigoid treatment before and after the introduction on omalizumab

		Patient 1	Patient 2	Patient 3	Patient 4	Patient 5
Age and sex		83, male	82, female	70, male	73, male	79, female
Date of diagnosis		2016	2019	2013	2011	2018
Treatment received before omalizumab		Topical and oral corticosteroids, antihistamines, doxycycline	Topical and oral corticosteroids, antihistamines	Topical and oral corticosteroids, antihistamines, azathioprine, methotrexate, colchicine, dapsone, mycophenolate mofetil, nbUVB phototherapy	Topical and oral corticosteroids, dapsone, antihistamines, methotrexate	Topical and oral corticosteroids, antihistamines, azathioprine, methotrexate
Comorbidities and previous surgeries		Type 2 diabetes, chronic hepatitis, hip replacement surgery	Type 2 diabetes, hypertension, osteoporosis	Type 2 diabetes, BPCO, BPH, persistent AF	Type 2 diabetes, BPH, vitiligo, atopic dermatitis, atrial fibrillation	Type 2 diabetes, HTN, hip replacement surgery, CKD stage III, dyslipidemia, grade I obesity
Number of injections		9	7	17	6	8
Follow‐up period		1 year, 8 months	9 months	3 years	8 months	1 year
ABSIS score	Before treatment	148	135	96	115,5	99
	During treatment	0	0	0	0	0
BSA%	Before treatment	99%	90%	64%	77%	66%
	During treatment	0%	0%	0%	0%	0%
Pruritus	Before treatment	VAS 8	VAS 8	VAS 10	VAS 10	VAS 9
	During treatment	VAS 3	VAS 6	VAS 0	VAS 3	VAS 0
Concurrent therapy given with omalizumab		Antihistamine, oral prednisone, topical betamethasone	Antihistamine, oral prednisone, topical betamethasone	Antihistamine, oral prednisone, topical desoxymethasone	Antihistamine, oral prednisone, topical betamethasone dipropionate	Antihistamine, oral prednisone
Diagnostic assay		H&E, DIF, IIF, SSS, Ab BP180, BP230, CollVII	H&E, DIF, Ab BP180, BP230,	H&E, DIF, IIF, SSS, Ab BP180, BP230, CollVII	H&E, DIF, Ab BP180, BP230	H&E, DIF, Ab BP180, BP230,

Abbreviations: ABSIS, autoimmune bullous skin disorder intensity score; BSA%, body surface area percentage involved; CS, corticosteroids; DIF, Direct immunofluorescence; H&E, Hematoxylin eosin histological examination; IIF, Indirect immunofluorescence; nbUVB, narrow‐band UVB; SSS, Salt‐split skin indirect immunofluorescence; VAS, visual analogue scale.

**TABLE 2:** Patient's bullous pemphigoid autoantibodies, IgE, and eosinophil titers before and during treatment with omalizumab

	BP180 ELISA (*N* < 20 U/ml)		BP230 ELISA (*N* < 20 U/ml)		IgE (*N* < 240 KU/I)		Eosinophilia (*N* = 0,1–0,5 ×10^3^/μl)	
	Before treatment	During treatment	Before treatment	During treatment	Before treatment	During treatment	Before treatment	During treatment
Patient 1	<20	<20	<20	<20	1638	3533	0,66	0,49
Patient 2	60.8	63.2	67.5	46.1	512	468	0,52	0,27
Patient 3	254,76	27.3	67,10	5.2	>5000	747	0,43	0,37
Patient 4	166.9	3.5	91,9	29.1	2061	2050	0,88	0,37
Patient 5	190,5	43.9	<20	<20	52	152	0,47	0,18

Abbreviation: ELISA: enzyme‐linked immunosorbent assay.

## 3 RESULTS

All the patients tolerated omalizumab without side‐effects. The mean duration of treatment was 9.2 months (median: 8 months, range: 12 months), while follow‐up lasted a mean of 17 months (median: 12 months, range: 28 months). Moreover, in all cases it was possible to reduce the dose of CS given to control the disease, in one case the patient could interrupt their use altogether. Furthermore, all the patients experienced complete resolution of their cutaneous lesions, and a reduction of pruritus, while two also manifested the complete resolution of the latter.

Three of the patients had a relapse of BP symptoms once omalizumab was discontinued at the end of a cycle, but soon recovered upon starting the following cycle.

The laboratory tests demonstrated a slight reduction in the titer of circulating IgE antibodies in two patients and a clear reduction in one. A reduction in anti‐BP180 and BP230 antibodies was demonstrated again in three cases, however, one of the patients presented with negative (<20 KU/L) titers for both autoantibodies at the time of diagnosis. Finally, the quantity of eosinophils in the patients' sera was reduced in four of the five patients evaluated.

## 4 CONCLUSIONS

BP is an autoimmune blistering disease, characterized by an immune response against BP180 and BP230. It mainly affects elderly patients and its incidence is growing in parallel with the general aging of the population. Clinically, it is usually a chronic disease with a waxing and waning course of exacerbations and regressions, which may last from months to years, associated with significant morbidity, which can severely affect quality of life. Mortality is high, ranging from 12% to 40% in the first year.^1^, ^2^ Complications have been found to arise secondary to treatment with systemic CS and immunosuppressive drugs, which are the mainstay of therapy. The latest European^10^ and Italian^11^ guidelines on the treatment of BP, described the primary goals of therapy as not only aimed at the treatment of the skin manifestations, pruritus and prevention of recurrences, but also underline the importance of improving the quality of life of patients, limiting the side‐effects related to the drugs employed, especially in the elderly.

Omalizumab, an anti‐IgE monoclonal antibody, has been included in the European, Italian, German^12^ guidelines for patients with treatment‐resistant BP, with a Grade‐4 recommendation. Furthermore, it was recently cited in the updated French 2021 guidelines,^10^ where it is listed as an option for the treatment of a subset of patients affected by corticosteroid‐dependent BP, an entity whose features preclude the possibility of gradually tapering the dosage of the systemic corticosteroids, thus exposing the patients to an increased risk of complications.

With the aim of reducing said risk, we studied the efficacy of omalizumab in reducing the dosage of systemic CS in the patients who received treatment in our clinic and we found that in 100% of cases it was possible to administer lower doses of steroids while controlling the disease. One of the patients could also continue with omalizumab monotherapy and discontinued systemic corticosteroids altogether. We observed complete remission of skin lesions and a decrease in itching in all five patients, while pruritus resolved in two cases. These findings suggest that omalizumab can be used as an adjuvant therapy in corticosteroid‐dependent BP. Among the laboratory results, we observed a marked decrease in IgE in one patient only. This can be explained by the fact that omalizumab prevents IgE binding to its receptor but it may prolong its half‐life in some patients by slowing its clearance by the kidneys. Patient three, who had the greatest reduction in IgE, was one of the first patients recruited and therefore subjected to therapy for the longest time (17 injections). The efficacy of omalizumab can be more clearly understood in the reduction of eosinophils, linked to a lower activity of IgE on mast cells.

These results should be seen in light of some limitations. First, the dosing protocol chosen for the administration of omalizumab was that indicated for chronic idiopathic urticaria. This choice stems from the fact that it is the only existing protocol for the administration of omalizumab in patients affected by dermatological diseases, as the only other approved indication for this drug is severe asthma. Consequently, the dosage and intervals of administration might not be the most appropriate for patients affected by BP, as we have observed by studying our patients, who, in 60% of cases, had a relapse during the 8‐week suspension period. This finding suggests that patients with BP treated with omalizumab should receive long‐term treatment with the monoclonal antibody, entailing economic consequences.

Second, all the patients included in our study had a long‐standing history of BP and had previously received various other treatments before beginning omalizumab. Thus, the empirical results reported herein should be considered in the light of the fact that patients who have a treatment‐naïve BP might respond differently to this therapy. Therefore, we believe that further studies evaluating the effects of this monoclonal antibody in the latter should be carried out.

## AUTHOR CONTRIBUTIONS

Camilla Vassallo, Anita Somenzi, Mara De Amici, Stefania Barruscotti, and Valeria Brazzelli contributed to the preparation and finalization of this article. All authors confirm the absence of conflict of interest.

## ETHICS STATEMENT

Informed consent was taken from all subjects.

## Data Availability

The data that support the findings of this study are available from the corresponding author upon reasonable request.
